# Heterosis goes underground

**DOI:** 10.1093/jxb/erab394

**Published:** 2021-09-03

**Authors:** Antonio J Monforte

**Affiliations:** Instituto de Biología Molecular y Celular de Plantas (IBMCP), Consejo Superior de Investigaciones Científicas (CSIC), Universitat Politècnica de València (UPV), Valencia, Spain

**Keywords:** Breeding, cucurbit, hybrid, rootstock, yield

## Abstract

This article comments on:

**Dafna A, Halperin I, Oren E, Isaacson T, Tzuri G, Meir A, Schaffer AA, Burger J, Tadmor Y, Buckler ES, Gur A. 2021**. Underground heterosis for yield improvement in melon. Journal of Experimental Botany 72, 6205–6218.

**Heterosis has been widely exploited in plant breeding since the beginning of the 20th century to obtain high-yielding cultivars. Dafna *et al.* (2021) studied the yield variation in a melon variety grafted onto a collection of hybrid rootstocks, demonstrating that yield heterosis can also be induced from the rootstock to the scion. The authors propose a new approach to exploit root potentialities instead of focusing on root architecture to investigate root-mediated plant performance. This approach is especially interesting in crops, such as melon, where the crosses between genetically highly divergent inbreds produce hybrids that do not maintain the quality parameters expected by growers and consumers**.

The development of hybrid cultivars derived from the cross between two inbred lines is a common approach in plant breeding since the release of the first maize hybrids in the early 20th century (Kinsgsbury, 2009). The success of hybrid varieties could be explained by two obvious causes: combination of genetic homogeneity with variability; and protection of breeder rights. Inbreds provide a homogenous response to agricultural practices, the management of the culture is easier for growers, and the products for industry and consumers are more homogeneous compared with open-pollinated (heterogeneous) cultivars. However, the lack of genetic variability of inbreds reduces their capacity to adapt to environmental changes. Hybrids combine genetic homogeneity with genetic variability so that they provide a homogeneous response and better adaptation to the environment. Moreover, hybrid seeds cannot be reproduced by growers, who need to obtain their seed stock from the suppliers, so that the breeder’s rights are guaranteed. However, there is another ‘magic’ reason: hybrids may overcome the yield and/or other agronomic traits of both inbred parents (Lippman *et al.*, 2007). We can imagine the surprise and excitement of early geneticists when they observed this so-called heterosis effect for the first time. Consequently, hybrid seed varieties were first developed for cereals after 1930 and later for horticultural crops such as tomato during the 1980s (Grandillo *et al.*, 1999). Nowadays, most modern cultivars are hybrids. Currently, 15–30% of the calories that we consume are attributed to the yield increase of hybrids (Duvick, 2001).

## Heterosis

According to the quantitative genetics theory, heterosis is defined as the difference between hybrid and inbred means (Falconer and Mackay, 1996). This definition refers to the ‘mid-parent heterosis’ (MPH); however, in applied breeding, the term heterosis is used to indicate that the hybrid overcomes the performance of any of the parents [best-parent heterosis (BPH), Box 1]. Classically, heterosis has been explained by three non-exclusive genetic causes (Box 1): (i) overdominance at a single gene, where the effects of a gene in the heterozygous state overcome the effects of both homozygotes; (ii) dominance complementation of slightly deleterious recessive alleles that are present in the inbred parents—this includes pseudo-overdominance, where two tightly linked genes complement each other but they are transmitted to the progeny as a single locus; and (iii) epistasis, where the new genetic interactions in the hybrid increase the trait. There are several examples of single locus overdominance such as, for example, the tomato *Flowering Locus T* (Krieger *et al.*, 2010); however, those seem to be more the exception than the rule. Heterosis is the result of multiple loci; thus, dominance complementation of multiple loci has been proposed as an important contributor to heterosis (Huang *et al.*, 2015) and this hypothesis has been adopted by a number of researchers (Schnable and Springer, 2013; Wallace *et al.*, 2018). Epistasis may have an important role, but most of the experimental designs have a low power to detect epistasis, so its possible contributions may be dismissed. In fact, recently, Xiao *et al.* (2021) using a new design with 42 840 F_1_ maize hybrids concluded that epistatic quantitative trait loci (QTLs) have an important contribution in hybrid heterosis. The molecular basis of heterosis is under debate: structural variations, gene dosage, and epigenetics have all been suggested to have an important role in heterosis (Liu *et al.*, 2020).

**Box 1. Different behaviour of hybrids and genetic basis of heterosis**

A hybrid from two inbred parents (P1 and P2) may show three different behaviours for a given trait relative to its parents’ performance: a phenotypic value equal to the mid-parent (MP) value (additive), a higher value than that of the MP but lower than that of the parents (mid-parent heterosis, MPH), or a higher value than any of the parents (best-parent heterosis, BPH). The genetic basis of heterosis according to the classical model can be explained by three non-exclusive causes: (i) overdominance, where the value of heterozygotes at a single locus (Aa) is superior to any of the homozygotes (AA or aa); (ii) dominance complementation, where inbreds contain different combinations of slightly deleterious recessive alleles (aa, bb) and the combination of several heterozygous loci (AaBb) surpasses both inbred values; and (iii) epistatic interactions, where new interactions arise (Aa×Bb) in the hybrid that increase the phenotypic value.

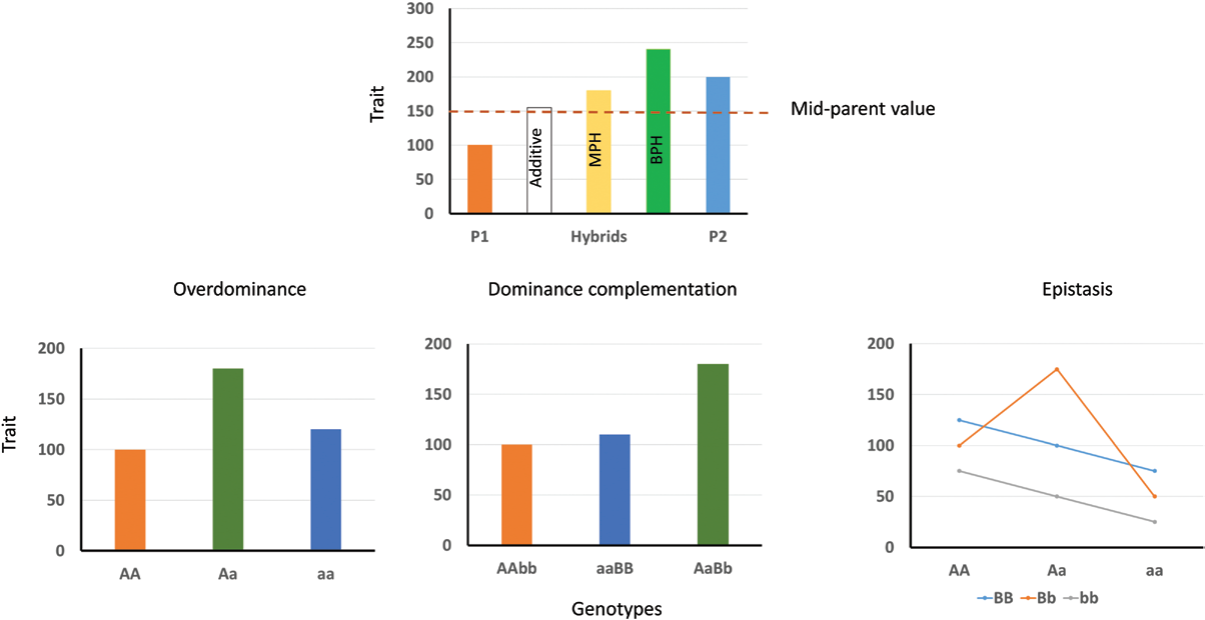

## Heterosis in melon

Heterosis has rarely been studied in melon. The few published works include a limited number of crosses between varieties, so general conclusions cannot be drawn (Monforte *et al.*, 2005; Napolitano *et al.*, 2020). Melon germplasm presents a high genetic diversity (Gonzalo *et al.*, 2019), but the exploitation of this variability for hybrid breeding has not been possible. Melon varieties are classified in horticultural groups or market classes according to defined—and usually incompatible from the consumers’ point of view—fruit quality characteristics (Pitrat, 2017). For example, cantaloupes are highly aromatic with a relatively short shelf-life, whereas ‘Piel de Sapo’ or honeydews are not aromatic with a long shelf-life. Hybrids between ‘Piel de Sapo’ and cantaloupes are less aromatic than cantaloupes and with a shorter shelf-life than ‘Piel de Sapo’, so the fruit quality characteristics are not adequate for their traditional market targets. Thus, hybrid seeds are produced only between inbreeds belonging to the same horticultural group, where the genetic diversity is relatively low.

Dafna *et al.* (2021) took an alternative approach to exploit heterosis in melon: instead of evaluating hybrids between genetically distinct varieties, they used 190 hybrids from crossing 20 highly diverse inbreds as rootstocks to evaluate the rootstock effect on agronomic traits in a common scion. Rootstocks are widely used in cucurbits and other crops to avoid soil-borne disease, but the effects of rootstock on agronomic traits have rarely been evaluated (Flores-León *et al.*, 2021). The results obtained by Dafna *et al.* (2021) are strikingly similar to those obtained with studies with hybrids performed in other crops: BPH was found for reproductive traits such as fruit number and total yield, as reported by Semel *et al.* (2006), while other morphological and metabolic traits such as fruit weight and soluble solid concentration (SSC), respectively, did not show general heterosis, as found by Monforte *et al.* (2005). Fruit quality was assessed as sugar content, finding no effect of the rootstocks on SSC. Even though fruit quality is also composed of traits other than SSC, such as volatile composition, texture, firmness, and post-harvest behaviour, and the fact these parameters can be affected by the rootstock (Flores-León *et al.*, 2021), the results are very promising to exploit the huge melon genetic diversity for hybrid breeding. Furthermore, a genome-wide association study found loci associated with root-mediated heterosis which opens up the possibility for molecular breeding and genomic prediction of rootstock hybrids.

## Perspectives

The approach proposed by the authors to study rootstock influences on scion agronomic performance instead of the direct study of root characteristics opens up new strategies for breeding. Rootstocks have been widely used to cope with biotic and abiotic soil-related stresses. The authors have demonstrated that this approach can also be used to improve agronomic traits and exploit heterosis. Further studies will determine the rootstock effects in a broader range of agronomic and fruit quality parameters, but these preliminary results are already very promising. Breeding can also go underground, by-passing barriers (including reproductive barriers) that hamper the use of some hybrids directly in cultivation. The detection of loci involved in root-mediated heterosis demonstrates the strong genetic determination of this phenomenon, opening up the path to develop genomic prediction to decide *a priori* the best hybrid combinations to test. Underground biology is coming to the fore. We can implement all the breeding, genetic, and genomics tools developed to date to raise the underground onto our dining tables. Time to turn on our flashlights and shed light on the underground.
